# Ex Vivo Oncolytic Virotherapy with Myxoma Virus Arms Multiple Allogeneic Bone Marrow Transplant Leukocytes to Enhance Graft versus Tumor

**DOI:** 10.1016/j.omto.2016.12.002

**Published:** 2016-12-14

**Authors:** Cameron L. Lilly, Nancy Y. Villa, Ana Lemos de Matos, Haider M. Ali, Jess-Karan S. Dhillon, Tom Hofland, Masmudur M. Rahman, Winnie Chan, Bjarne Bogen, Christopher Cogle, Grant McFadden

**Affiliations:** 1 Molecular Genetics and Microbiology, University of Florida, Gainesville, FL 32611, USA; 2 College of Agriculture and Life Sciences, University of Florida, Gainesville, FL 32611, USA; 3 Department of Experimental Immunology, Academic Medical Center, Amsterdam, 1105 the Netherlands; 4 DNAtrix, Inc., Houston, TX 77021, USA; 5 Centre for Immune Regulation, Institute of Immunology, University of Oslo and Oslo University Hospital, 0313 Oslo, Norway; 6 KG Jebsen Centre for Influenza Vaccine Research, University of Oslo, 0313 Oslo, Norway; 7 Department of Medicine, University of Florida, Gainesville, FL 32611, USA

**Keywords:** myxoma virus, multiple myeloma, MOPC315.BM, allogenic transplant, neutrophils, T cells

## Abstract

Allogeneic stem cell transplant-derived T cells have the potential to seek and eliminate sites of residual cancer that escaped primary therapy. Oncolytic myxoma virus (MYXV) exhibits potent anti-cancer efficacy against human cancers like multiple myeloma (MM) and can arm transplant-derived T cells to become more effective cancer killers in vitro and in an immunodeficient xenotransplant murine model. Here, we tested ex vivo MYXV virotherapy against residual murine MM in immunocompetent mice using an allogeneic mouse-mouse model. In contrast to all human MM cell lines previously tested, the murine MM cell line tested here was highly resistant to direct MYXV infection and oncolysis in vitro. Despite this in vitro resistance, we found that ex vivo MYXV-armed allogeneic bone marrow (BM) transplantation dramatically ablated pre-seeded residual MM in vivo. Unexpectedly, we show that both neutrophils and activated T cells from the donor function as virus-armed carrier cells, and MYXV-preloaded cells enhanced MM killing. Our results demonstrate a novel therapeutic paradigm for residual cancer, in which multiple classes of allotransplant leukocytes can be armed by MYXV ex vivo to enhance the graft-versus-tumor effects.

## Introduction

Current therapeutic regimens for many blood cancers involve high-dose myeloablative therapy, followed by transplantation of hematopoietic stem cells (HSCs) to reconstitute the patient’s normal immune functions.^1^, ^2^, ^3^, ^4^, ^5^ Despite aggressive and ever-evolving therapeutics, relapse rates for many of these cancers remain high, largely because of residual disease that escapes myeloablative treatment.^6^, ^7^ Transplantation of HSCs from mobilized peripheral blood mononuclear cells (PBMCs) or bone marrow (BM) following a conditioning regimen can either be accomplished by an autologous graft, where the patient donates BM or stem cells prior to chemotherapy or radiotherapy, or as an allogeneic graft, in which a human leukocyte antigen (HLA)-matched donor provides the HSCs that will repopulate the recipient immune system.^8^ Allotransplantation carries the added benefit of a graft-versus-tumor (GVT) response, where T cells from the donor transplant can traffic to and recognize foreign cancer cells in situ and kill the disseminated disease that escaped traditional treatment.^9^, ^10^ This added GVT benefit, however, is accompanied by the significant risk of alloreactive T cells recognizing normal host tissues in the same fashion, leading to graft-versus-host disease (GVHD),^11^ a cause of significant morbidity and mortality. Thus, therapeutic strategies that accentuate the GVT response of an allotransplant, although rendering them safer for GVHD, would open new treatment options to eliminate residual cancer cells after myeloablative therapy.

Myxoma virus (MYXV) is an oncolytic poxvirus that is tightly host restricted to rabbits in nature.^12^ Importantly, MYXV has the capacity to target and kill many types of human cancer cells.^13^, ^14^ In fact, MYXV can directly infect and kill human multiple myeloma (MM) cell lines as well as primary patient bone marrow samples contaminated with MM. Notably, MYXV selectively deletes MM cells from PBMCs or BM from patients ex vivo without harming CD34^+^ HSCs because normal stem cells are incapable of binding MYXV.^15^, ^16^, ^17^, ^18^, ^19^ The fate of MYXV-infected human MM cells is a rapidly induced apoptosis that is triggered through activation of the extrinsic caspase-8 pathway.^20^ Previous studies investigating MYXV purging of human MM engrafted into irradiated NOD/Scid/IL2Rγ^−/−^ (NSG) mice demonstrated that ex vivo pre-treatment of an allo-HSC transplant with MYXV maintains GVT effects in vivo without altering the engraftment of normal human HSCs into the recipients.^15^ In addition to this, ex vivo virotherapy with MYXV also completely eliminated acute GVHD in this xenotransplanted mouse model by suppressing the subsequent expansion and infiltration of alloreactive human T lymphocytes into normal recipient tissues.^16^ Recently, it was shown that normal human T cells can bind MYXV and can then co-traffic with these cells, but the virus infection is launched only after T cell activation.^21^ Importantly, activated and/or infected T cells can subsequently donate the oncolytic MYXV to human MM cells via cell-cell contact.^21^

Although animal experiments investigating ex vivo virotherapy with MYXV against MM have previously been performed using human HSCs and myeloma samples xenografted into highly immunodeficient NSG mice, we next investigated a classical allogeneic mouse-to-mouse model. Thus, we tested the potential for ex vivo MYXV virotherapy to eliminate pre-seeded residual disease in an immunocompetent murine model of MM by exploiting tagged murine MOPC315.BM cells.^22^, ^23^ MOPC315.BM cells are a murine plasmacytoma cell line that can engraft into syngeneic immunocompetent BALB/c mice and cause a progressive disease highly recapitulative of human MM, including BM invasion, osteolysis, and paralysis.^22^, ^23^ We report that, in stark contrast to all human MM tested, this murine MOPC315.BM cell line is resistant to direct infection with MYXV because of a defect in virus binding to these cells. Despite this in vitro resistance to free virus, ex vivo MYXV virotherapy of a donor murine C57BL/6 allograft, after transplantation into BALB/c mice bearing pre-seeded MOPC315.BM, nearly completely ablated the residual myeloma burden in the recipients. Unexpectedly, when individual cell classes in the donor BM allotransplant were tested for the ability to kill target MM in a virus-dependent fashion, both activated T cells and neutrophils within the C57BL/6 allotransplant were able to induce MYXV-enhanced cell killing of MOPC315.BM. Taken together, our experiments demonstrate that both neutrophils and activated T cells in BM transplant samples have the therapeutic potential to act as carrier cells after ex vivo oncolytic virotherapy with MYXV for allotransplantation to target and eliminate residual cancer.

## Results

### Mouse MOPC315.BM Cells Are Highly Resistant to Direct Infection with MYXV

**Figure 1:**
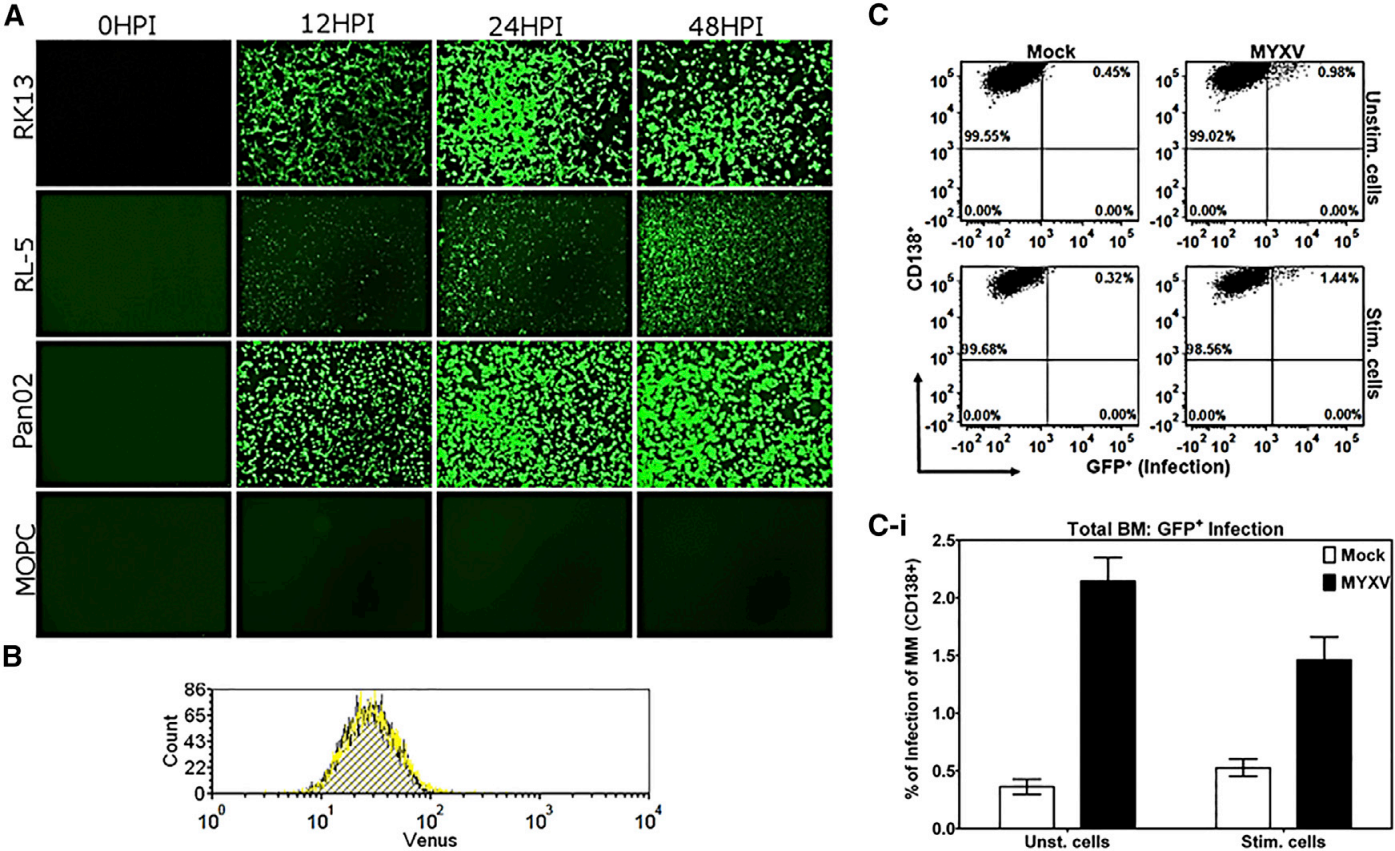
Myxoma Virus Infection of MOPC315.BM Cells (A) vMyx-M135KO-GFP (MOI of 10) infection of rabbit epithelial (RK13) and T cells (RL-5), mouse pancreatic cancer cells (Pan02), and mouse MOPC315.BM DsRed cells was compared in a time course. (B) MOPC315.BM Luc cells were assayed for surface virus binding comparing mock (blue histogram) with cells incubated with vMyx-Venus/M093 virus (yellow histogram). (C and Ci) Flow cytometric analysis of MOPC315.BM DsRed cells mock-treated or infected with vMyx-M135KO-GFP at MOI of 10 with or without cell stimulation with PMA and ionomycin. (Ci) Shown is the mean ± SEM of at least three independent experiments.

To characterize the MOPC315.BM model of immunocompetent murine MM, we first tested the cultured MM cells to determine whether, like human MM, they were permissive to MYXV infection (Figure 1). MOPC315.BM cells tagged with DsRed were compared in parallel with three control cell lines known to be permissive for MYXV infection: RK13 and RL5 are permissive rabbit epithelial and T cell lines, respectively,^24^ and Pan02 is a murine pancreatic cancer cell line that is fully permissive for MYXV.^25^ Cells were infected with vMyx-M135KO-GFP at a multiplicity of infection (MOI) of 10 focus-forming units (ffu)/cell. vMyx-M135KO-GFP is a leading clinical candidate construct that is nonpathogenic to rabbits but exhibits improved oncolytic potential against human cancer cells,^13^, ^26^ and that expresses GFP as a reporter gene under a synthetic early or late viral promoter. Infection was monitored over the course of 48 hr (Figure 1A). In this experiment, control RK13, RL5, and Pan02 cells all accumulated GFP expression over time, indicating that viral gene expression is progressing. Strikingly, MOPC315.BM DsRed cells exhibited no detectable GFP expression whatsoever, suggesting that infection was either aborted or prevented at some early stage.

To assess at which stage of infection MOPC315.BM cells were blocked, we next tested whether MYXV virions could bind to the cell surface of MOPC315.BM cells. The cell binding assays were performed with a MYXV construct that incorporates the Venus fluorescent protein fused to a virion component, vMyx-M093/Venus.^27^ MOPC315.BM cells were either mock treated with PBS or incubated with vMyx-M093/Venus at an MOI of 20 at 4°C for 1 hr. Unbound virus was washed twice with PBS + 5% fetal bovine serum (FBS). Flow cytometry was used to assess Venus-tagged virus binding (Figure 1B). Mock-treated cells (blue histogram) were compared with those labeled with virus (yellow histogram). No change in fluorescence was detected between the mock- and virus-treated cells, indicating that the failure of MYXV to infect MOPC315.BM cells in culture is because of the inability of the virus particles to bind to these cells.

For some target cell lines, the susceptibility to MYXV oncolysis is dependent on cell stimulation.^21^, ^28^ To test whether MOPC315.BM might display a stimulation-dependent permissiveness, we either mock-treated or infected MOPC315.BM cells with vMyx-M135KO-GFP with or without stimulation with phorbol 12-myristate13-acetate (PMA) and ionomycin. After 48 hr, the levels of virus infection were determined using flow cytometry (Figures 1C and 1Ci). In the absence of stimulation, infection resulted in a minimal increase in GFP signal (mean ± SE: 2.144% ± 0.205%), and stimulation of MOPC315.BM cells did not increase the GFP signal (mean ± SE: 1.500% ± 0.200%). This indicates that mouse MOPC315.BM cells, unlike human MM cells, are highly resistant to direct infection by free MYXV in culture. Although for most oncolytic models this in vitro resistance would preclude further testing of MYXV against this cancer type in vivo, it was previously shown that virus-infected and/or -activated human T cells can transmit MYXV to target cancer cells in a cell-cell contact fashion.^21^ Thus, this MOPC315.BM model of residual cancer can specifically query the direct effects of ex vivo MYXV virotherapy on GVT efficiency following an allotransplant, using an oncolytic virus that cannot directly infect or kill the target cancer cells via free virus.

### Ex Vivo MYXV Virotherapy of an Allograft Increases Survival and Reduces Pre-existing Residual Myeloma Burden

**Figure 2:**
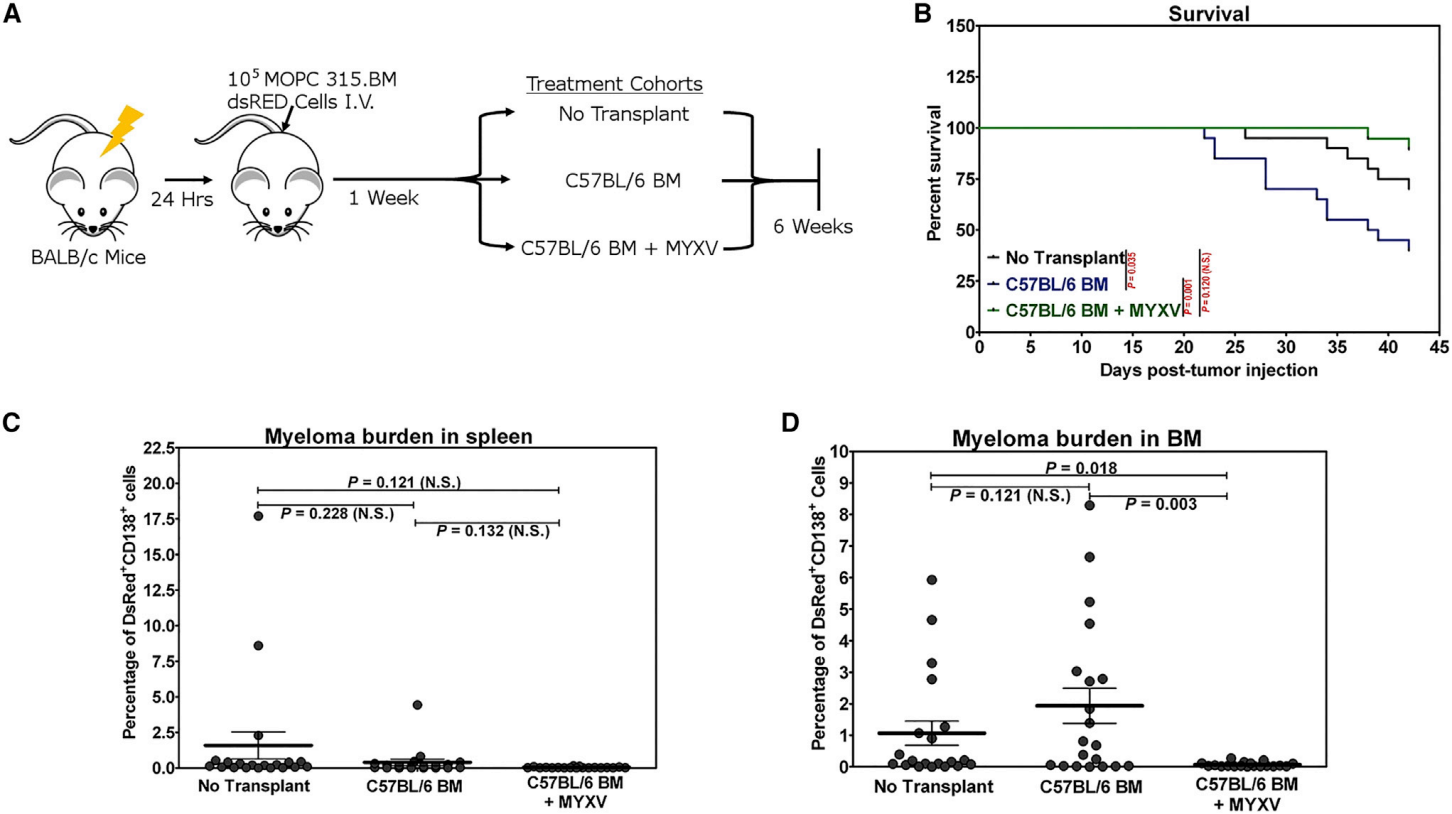
Ex Vivo MYXV Virotherapy of an Allogeneic BM Transplant against Residual MOPC315.BM Myeloma Burden (A) The experimental workflow is outlined. (B) Survival of animals was monitored following establishment of residual MOPC315.BM DsRed burden and treating with either no transplant (n = 20), untreated BM transplant from C57BL/6 mice (n = 20), or BM transplant from C57BL/6 mice pre-treated ex vivo with vMyx-135KO-EGFP for 1 hr at MOI of 10 (n = 19). (C and D) Myeloma burden was measured in the spleen (C) or BM (D) of the studied animals using flow cytometry to quantify percentage of total cells that are CD138^+^, DsRed^+^ MOPC315.BM DsRed cells. Significance in survival and tumor burden were determined using the log rank test and the Student t test, respectively. p < 0.05 is deemed significant. N.S., not significant.

Despite the observed resistance to MYXV infection in vitro, we next sought to determine whether carrier cells in murine BM could ferry MYXV to the site of tumor burden and facilitate elimination of low levels of pre-existing residual myeloma disease, and thus test directly whether MYXV could enhance GVT. The in vivo experimental design is outlined in Figure 2A. In brief, 1 × 10^5^ MOPC315.BM DsRed cells were delivered intravenously to BALB/c mice to establish minimal systemic disease; then 1 week later, mice were either administered no transplant (n = 20: cohort I), an allotransplant of 2 × 10^6^ cells of whole C57BL/6 BM (n = 20: cohort II), or 2 × 10^6^ cells of C57BL/6 BM pre-treated ex vivo for 1 hr with vMyx-135KO-GFP prior to infusion (n = 19: cohort III). When the recipients reached endpoint criteria as described in the Materials and Methods, or after 6 weeks, they were euthanized, necropsy was performed to assess levels of metastasis, and spleen and BM were harvested for quantitative analysis of CD138^+^ DsRed^+^ myeloma burden. Over the course of the experiment, 30% of control cohort I (no transplant) recipients reached endpoint criteria because of progressive myeloma disease. Survival of animals in the study is shown in Figure 2B (p = 0.035, no transplant versus C57BL/6 BM; p = 0.001, C57BL/6 BM versus C57BL/6 BM + MYXV; p = 0.120 [not significant (N.S.)], no transplant versus C57BL/6 BM + MYXV according to the log rank test). Animals receiving uninfected C57BL/6 BM, however, demonstrated a marked increase in morbidity (60% of the animals) over the course of the study, likely because of both progressing MM disease as well as allograft-induced GVHD. However, treatment with the equivalent allotransplant, which was pre-incubated for 1 hr ex vivo with MYXV prior to infusion, exhibited reduced morbidity, with only 10.5% of the animals in the study succumbing during the 6-week study. Myeloma burden of animals in the study was next assessed in the spleen (Figure 2C) and BM (Figure 2D) by flow cytometry. A slight reduction of myeloma burden was observed in the spleen when we compared the mean ± SEM of no-transplant controls (1.661% ± 0.999%) with animals receiving C57BL/6 BM alone 0.389% ± 0.223% (p = 0.228, N.S.). However, in BM, the myeloma burden was essentially unchanged (no transplant versus C57BL/6 BM: 1.123% ± 0.402% versus 2.038% ± 0.575%; p = 0.121, N.S.). However, ex vivo MYXV treatment of the allotransplant resulted in a consistent reduction in myeloma burden at the 6-week sacrifice point, and the majority of the animals manifested no myeloma burden as defined by the limits of FACS detection (e.g., 17/19 in spleen, 0.037% ± 0.010%, p = 0.121 for no transplant versus C57BL/6 BM + MYXV, p = 0.132 for C57BL/6 BM versus C57BL/6 BM + MYXV; 16/19 in BM 0.068% ± 0.017%, p = 0.018 for no transplant versus C57BL/6 BM + MYXV, p = 0.003 for C57BL/6 BM versus C57BL/6 BM + MYXV). Animals were scored as “disease-free” if they exhibited less than 0.1% DsRed^+^, CD138^+^ myeloma burden and had no detectable metastasis (Table 1). The in vivo data demonstrated that MYXV ex vivo treatment of an allotransplant greatly enhanced GVT against pre-seeded myeloma, which results in an improved clinical outcome and dramatically reduced myeloma burden despite the observed in vitro resistance of this MM line to direct oncolysis by free virus.

### Allotransplant Neutrophils Can Function as Novel Carrier Effector Cells for MYXV against Residual Myeloma

**Figure 3:**
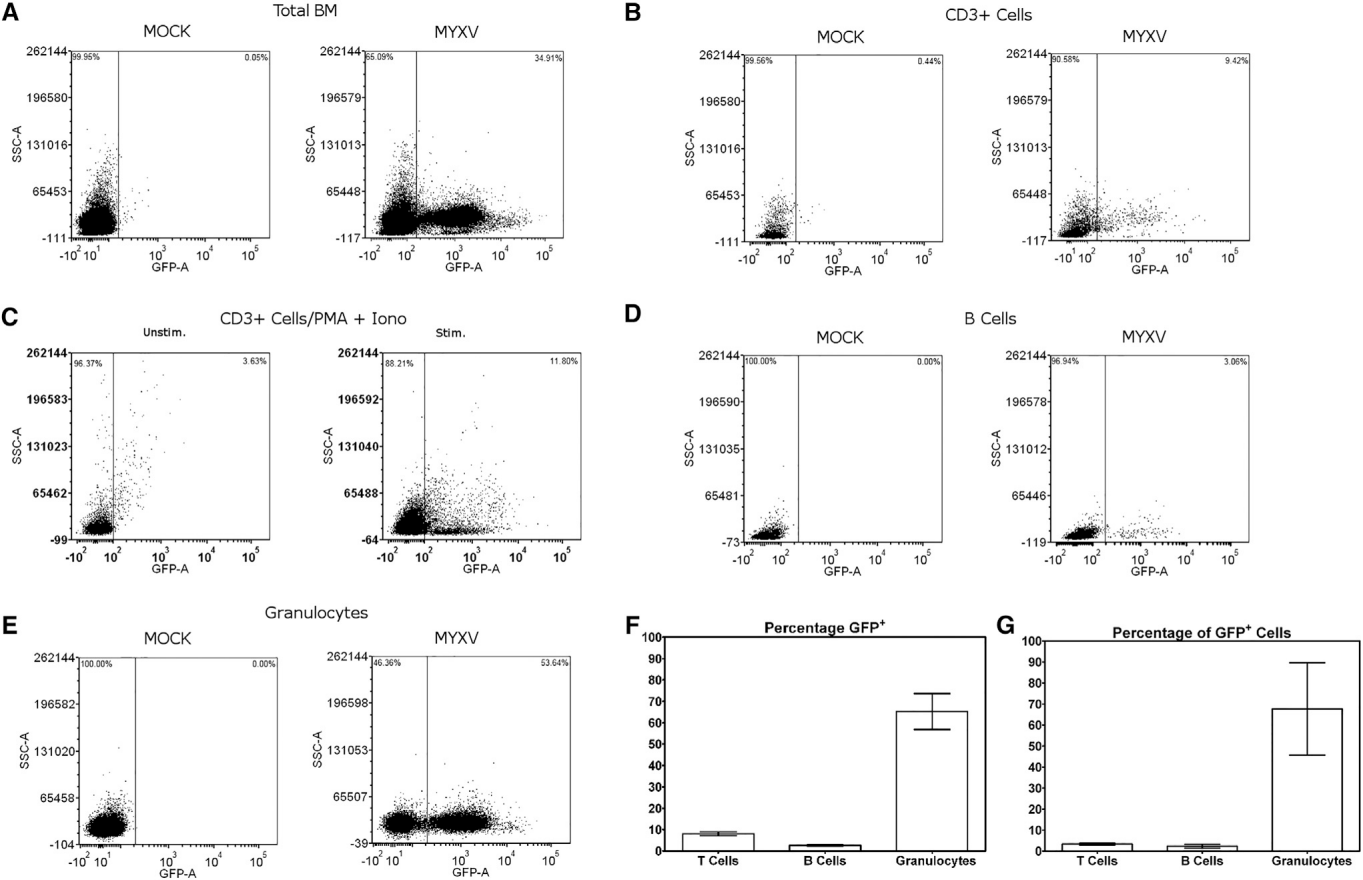
Infection of BM-Derived Murine Neutrophils and T Cells by Myxoma Virus To identify potential effector cells from the ex-vivo-treated transplant material, we infected BM from C57BL/6 mice with vMyx-M135KO-GFP at MOI of 10 for 12 hr, then stained for various cell surface markers and analyzed with flow cytometry for GFP^+^ cells. (A) Corresponds to whole BM. (B) BM-derived T cells were analyzed by CD3 staining. (C) Negatively selected BM-derived T cells were infected with vMyx-M135KO-GFP with or without stimulation with PMA/ionomycin and analyzed for GFP expression using flow cytometry. (D and E) Within the infected BM, B cells were identified by B220/CD19 staining (D), and granulocytes were identified as CD11b^+^/Ly-6B.2^+^/Ly-6G^hi^ (E). (F and G) Average percentage of cell populations that are GFP^+^ are graphed in (F) and percentages of the total GFP^+^ cells represented by different cell subtypes are graphed in (G); the mean of three independent experiments is shown as the height of the bar, and error bars represent the SE. The p values were determined using the Student’s t test. p < 0.05 was considered statistically significant.

Our in vivo experiments demonstrated that donor C57BL/6 BM contains cells whose anti-myeloma effects are enhanced by the ex vivo MYXV treatment. To identify potential virus carrier cells within C57BL/6 BM that might contribute to the elimination of residual MM, whole donor C57BL/6 BM was first cleared by RBC lysis and then infected with MYXV for 12 hr and subjected to analysis by flow cytometry to identify any MYXV-permissive cells (Figure 3). In total BM, virus-derived GFP was readily detectable in a subset of cells, indicating that subsets of MYXV-sensitive cells existed within the BM (Figure 3A). By using discriminatory cell surface markers, we identified and analyzed T cells, B cells, and granulocytes for the capacity to initiate vMyx-M135KO-GFP infection (GFP^+^) using flow cytometry. The mean ± SE are shown in Figure 3F (8.037% ± 0.919% T cells, 2.625% ± 0.435% B cells, 65.190% ± 8.387% granulocytes).The percentage of total GFP^+^ cells (MYXV-infected cells) and the corresponding distribution along each subpopulation is shown in Figure 3G (3.333% ± 0.467% T cells, 2.333% ± 0.906% B cells, 67.670% ± 22.000% granulocytes). Previous work has identified activated human T cells as effector and carrier cells for MYXV against human MM, because these cells were permissive for MYXV in a stimulation-dependent manner.^21^ Unstimulated murine T cells, when analyzed as a subset of the BM population, revealed only an average of 8.0% of total CD3^+^ cells as being GFP^+^ following infection (Figure 3B), and these T cells comprised only ∼3.3% of the total number of GFP^+^ cells (Figure 3G). Isolated murine CD3^+^ T cells also demonstrated a small increase of GFP, to 11.8% in infectivity when activated with PMA and ionomycin (Figure 3C), in stark contrast to the much higher levels of virus infection for activated human T cells.^21^ Murine B cells also displayed similar low levels of GFP^+^ signal, with an average of 9.55% of B cells becoming GFP^+^ (Figure 3F), which accounted for 2.3% of the total GFP^+^ cells in the infection (Figure 3G). Surprisingly, granulocytes from the MYXV-infected murine BM population were found to be readily susceptible for MYXV infection: 65.2% of granulocytes were GFP^+^ (Figure 3F), accounting for 67.7% of the total GFP^+^ cells in the murine BM (Figure 3G).

### MYXV Arms Stimulated BM-Derived Neutrophils to More Effectively Kill MOPC315.BM Cells

**Figure 4:**
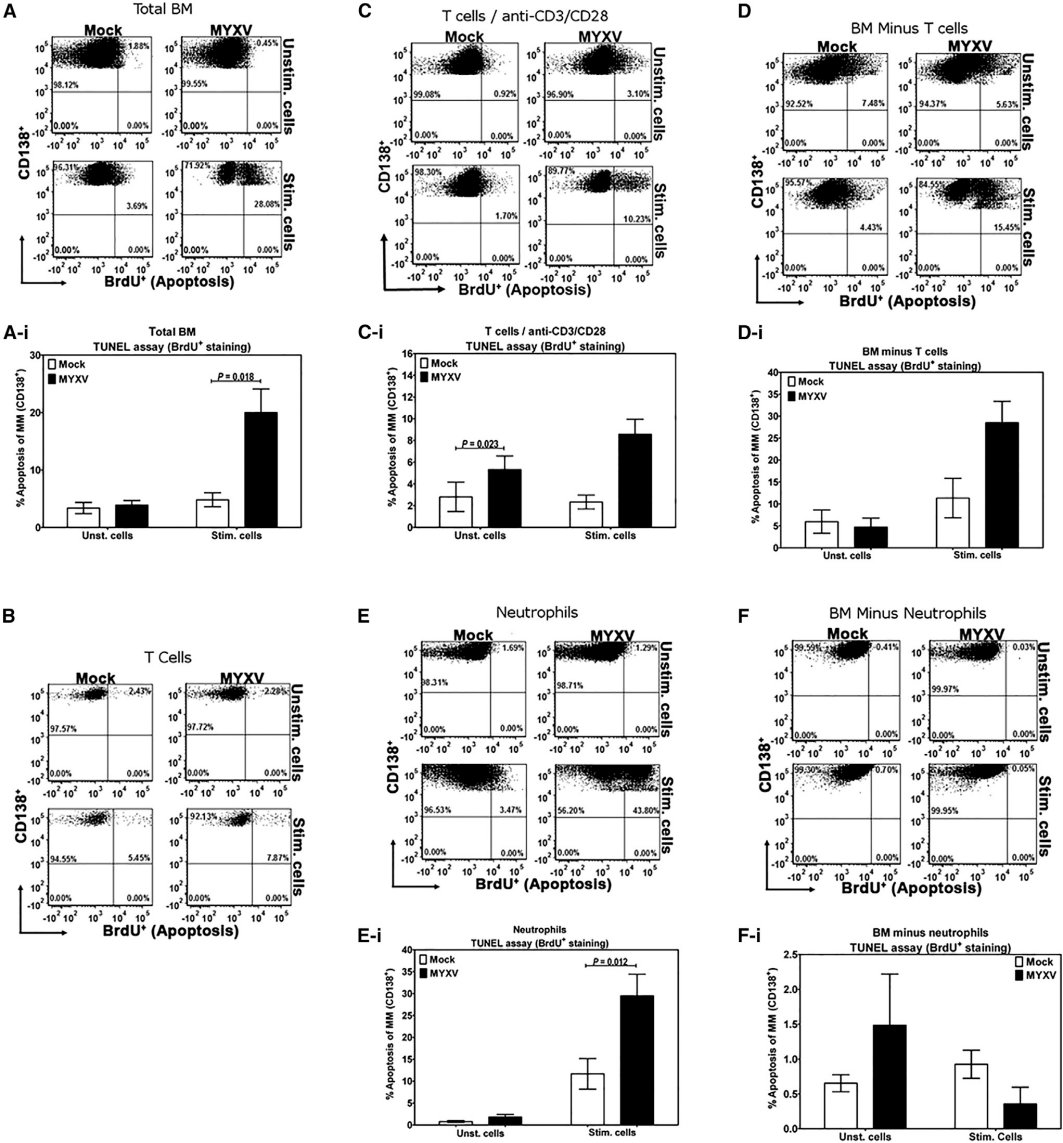
Apoptosis of MOPC315.BM Myeloma Cells Induced by Mock- or MYXV-Treated, Unstimulated, or Stimulated Murine Neutrophils and T Cells TUNEL assay followed by flow cytometric analysis were performed on admixtures of MOPC315.BM DsRed cells with C57BL/6 BM, or C57BL/6 BM-derived lymphocyte cells, at a ratio of 10:1 effector versus target cells, with or without cell stimulation, with or without vMyx-135KO-GFP. Co-culture of MOPC315.BM with whole BM (A and Ai), negatively selected T cells (B, C, and Ci) or T-cell-depleted cell fraction (D and Di), negatively selected neutrophils (E and Ei), and neutrophil-depleted fraction (F and Fi). Stimulation for all panels was performed with PMA/ionomycin except (C) and (Ci), in which T cells were stimulated with anti-CD3/CD28 Dynabeads. Results shown are representative of at least three independent experiments. The mean of at least three independent experiments is shown as the height of the bar, and error bars represent the SE. The Student’s t test was used to determine the p values. p < 0.05 was considered statistically significant.

To investigate effector roles of BM-derived cell subpopulations capable of harboring MYXV replication in the GVT response, we performed co-cultures of MOPC315.BM cells and either primary C57BL/6 BM or isolated C57BL/6 BM-derived T cells or neutrophils in the presence or absence of MYXV and with or without cell stimulation. From whole primary C57BL/6 BM, CD3^+^ T cells or neutrophils were isolated and then either mock treated or infected with MYXV at MOI of 10, washed, and then mixed with target MOPC 315.BM cells in the presence or absence of PMA/ionomycin stimulation. Cell viability (Figure S2) and apoptosis (Figure 4) were then measured in the target MM cells by flow cytometry. The isolated whole C57BL/6 BM was capable of inducing apoptosis in target MOPC315.BM myeloma cells only following PMA/ionomycin stimulation, but this killing was greatly enhanced in the presence of MYXV (Figures 4A and 4Ai) (mean ± SE of unstimulated cells: 3.388% ± 0.969% mock versus 3.902% ± 0.809% MYXV, p = 0.598 [N.S.]; mean ± SE of stimulated cells: 4.825% ± 1.222% mock versus 20.01% ± 4.084% MYXV, p = 0.018). Likewise, stimulation and MYXV infection also induced MM cell death compared with stimulated and mock-treated cells (Figures S2A and S2Ai) (mean ± SE of unstimulated cells: 10.360% ± 1.641% mock versus 11.250% ± 1.330% MYXV, p = 0.737 [N.S.]; mean ± SE of stimulated cells: 8.980% ± 1.587% mock versus 16.730% ± 2.530% MYXV, p = 0.044). No changes in the levels of apoptosis of MOPC315.BM myeloma cells were observed upon incubation with isolated C57BL/6 BM-derived CD3^+^ T cells that were either mock- or MYXV-treated and with or without PMA/ionomycin stimulation (Figure 4B, one representative experiment), but the stimulation of the donor T cells with anti-CD3/CD28 Dynabeads resulted in more target cell death than stimulation with PMA/ionomycin (Figures 4C and 4Ci; apoptosis of MM, mean ± SE of unstimulated cells: 2.810% ± 1.348% mock versus 5.310% ± 1.262% MYXV, p = 0.023; mean ± SE of stimulated cells: 3.807% ± 1.512% mock versus 8.577% ± 1.370% MYXV, p = 0.152 [N.S.]) (Figures S2C and S2Ci; MM cell death, mean ± SE of unstimulated cells: 1.603% ± 0.440% mock versus 1.743% ± 0.591% MYXV, p = 0.463 [N.S.]; mean ± SE of stimulated cells: 4.987% ± 0.0.843% mock versus 6.787% ± 0.494% MYXV, p = 0.298 [N.S.]). However, the BM cell fraction remaining after T cell removal (depleted fraction) still contained considerable capacity to induce cell death in the target MM cells in the presence of MYXV and cell stimulation (Figures 4D and 4Di; apoptosis of MM, mean ± SE of unstimulated cells: 5.973% ± 2.652% mock versus 4.703% ± 2.071% MYXV, p = 0.177 [N.S.]; mean ± SE of stimulated cells: 11.330% ± 4.501% mock versus 28.490% ± 4.902% MYXV, p = 0.095 [N.S.]) (Figures S2D and S2Di; MM cell death, mean ± SE of unstimulated cells: 11.770% ± 2.789% mock versus 13.690% ± 0.624% MYXV, p = 0.571 [N.S.]; mean ± SE of stimulated cells: 5.887% ± 0.918% mock versus 18.680% ± 4.844% MYXV, p = 0.152 [N.S.]). Because we had identified neutrophils as uniquely permissive for MYXV infection, we then tested the ability of purified neutrophils to kill MOPC315.BM cells (Figures 4E and 4Ei; apoptosis of MM, mean ± SE of unstimulated cells: 0.762% ± 0.238% mock versus 1.792% ± 0.604% MYXV, p = 0.185 [N.S.]; mean ± SE of stimulated cells: 11.670% ± 3.493% mock versus 29.500% ± 4.932% MYXV, p = 0.012) (Figures S2E and S2Ei; MM cell death, mean ± SE of unstimulated cells: 5.842% ± 1.415% mock versus 6.875% ± 2.044% MYXV, p = 0.649 [N.S.]; mean ± SE of stimulated cells: 3.354% ± 0.684% mock versus 7.740% ± 1.109% MYXV, p = 0.043). Unstimulated neutrophils (with or without MYXV) were essentially unable to induce MM cell death in MOPC315.BM cells, and PMA/ionomycin stimulation alone mediated only a small increase in apoptotic MM cells. However, in the presence of stimulation with PMA/ionomycin and MYXV, isolated neutrophils potently induced cell death in the target MOPC315.BM cells, and this MM killing capacity was essentially absent in the neutrophil-depleted BM fraction (Figures 4F and 4Fi; apoptosis of MM, mean ± SE of unstimulated cells: 0.653% ± 0.122% mock versus 1.483% ± 0.735% MYXV, p = 0.852 [N.S.]; mean ± SE of stimulated cells: 0.925% ± 0.202% mock versus 0.355% ± 0.242% MYXV, p = 0.176 [N.S.]) (Figures S2F and S2Fi; MM cell death, mean ± SE of unstimulated cells: 8.523% ± 1.086% mock versus 18.040% ± 5.763% MYXV, p = 0.389 [N.S.]; mean ± SE of stimulated cells: 12.730% ± 3.308% mock versus 13.550% ± 4.520% MYXV, p = 0.809 [N.S.]).

## Discussion

MYXV exhibits potent oncolytic potential against many human cancers, including MM, both in vitro and in vivo.^15^ Work to date on MYXV-mediated oncolysis of human MM has well characterized its ability to infect and kill human MM via a rapid induction of caspase-8-mediated apoptosis.^20^ In stark contrast to this MYXV sensitivity of human MM, we show here that the murine MM cell line MOPC315.BM is uniquely resistant to infection by free MYXV because of its inability to bind MYXV virions, indicating that these cells are highly resistant to direct virus oncolysis, at least as assessed in vitro. In previous reports investigating MYXV oncolysis of primary human acute myeloid leukemia (AML) cells, cell surface binding by virus was shown to be a major determinant of therapeutic benefit when preventing tumor engraftment into irradiated NSG mice.^28^ However, here we demonstrate that ex vivo virotherapy with MYXV arms cells within a donor allotransplant resulting in a dramatic positive enhancement of GVT in a model where the pre-seeded residual target tumor cell is highly resistant to direct infection by free virus. This represents a dramatic paradigm shift in oncolytic virotherapy, because cancer cells that are nonpermissive to the test virus in vitro were previously considered to be poor therapeutic candidates for that same virus in vivo. Instead, when tumor-homing leukocytes are loaded with virus ex vivo, even “virus-resistant” cancers can become susceptible to oncolytic virotherapy.

Allogeneic transplantation was exploited in this study, despite autotransplantation being the primary strategy for human MM patients,^5^ because we postulated that post-transplant activation of MYXV-loaded T cells would play a major role in the therapeutic benefits against residual cancer. Indeed, we observed dramatically improved reductions in residual disease burdens following allotransplantation with MYXV-treated C57BL/6 BM, suggesting that ex vivo virotherapy treatment may be an effective way to enhance the GVT effects of an allotransplant. Moreover, we believe these results demonstrate a capacity for targeted, carrier-cell-mediated clearance of residual tumor burden that has the potential to translate into treatment for any cancerous malignancy for which an allotransplant is performed. Furthermore, if ex vivo MYXV virotherapy also alleviates GVHD in humans as extensively as it does for human BM xenotransplantation into immunodeficient mice,^16^ it may even broaden the spectrum of malignancies for which an allotransplant can be performed and increase the allograft’s effectiveness at eradicating residual disease in general. A hallmark of this study is the ability of ex vivo MYXV virotherapy to reduce or eliminate disease progression when the transplant is delivered, at a time when the cancer burden is at the lowest possible level.

Human T cells have been recently investigated for carrier-cell-mediated oncolysis of MM using MYXV. Human T cells possess the capacity to ferry and deliver MYXV to human MM, and MYXV has been shown to enhance T-cell-mediated killing of human MM via cell-cell contact in an activation-dependent manner.^21^ Primary murine T cells, in this study, did demonstrate some ability for MYXV-enhanced cell killing of MOPC315.BM target cells, but not to the same robust extent as observed for MYXV-armed human T cells. Instead, the ability of MYXV-infected and/or -activated murine T cells to kill target MM cells was relatively minor compared with the dramatic levels of myeloma cell death induction mediated by MYXV-infected and/or -activated BM-derived neutrophils. This result was unexpected because T cells are believed to be the major effectors that mediate GVT following allo-transplantation. In addition to T cells, alloreactive natural killer (NK) cells have also started to be recognized as mediators of GVT.^29^

To our knowledge, although it is known that neutrophils are capable of efficiently infiltrating tumor tissues, this is the first report to identify neutrophils as an effective virus-carrier cell population for oncolytic virotherapy. However, to establish the relevancy of these new observations, the allogeneic murine model needs further investigation in order to ascertain whether neutrophils loaded ex vivo with virus can per se efficiently carry and deliver MYXV to directly target MM cells, thereby increasing the GVT effects. Alternatively, neutrophils loaded with MYXV may play other potentially indirect roles, for example, by interacting and activating natural killer (NK) cells to increase GVT.^30^ This latter possibility is reasonable because there is accumulating evidence showing that neutrophils can differentially switch phenotypes and manifest distinct subpopulations under different microenvironments. In this context, neutrophils can produce a large variety of cytokines and chemokines upon stimulation that could contribute to the increased anti-cancer effects we observed in this study. Moreover, neutrophils can also directly interact with dendritic cells (DCs), macrophages, NK cells, T cells, and B cells, so as to either potentiate or down-modulate both innate and adaptive immunity (reviewed in Yang et al.^31^). Therefore, future in vivo and in vitro studies will be needed to clarify the role of licensing NK cells on virus-augmented GVT effects using a classical allogeneic murine model. As well, the potential role of virus-loaded human neutrophils toward GVT also merits investigation.

Many studies have revealed a dual role of neutrophils in cancer biology.^32^, ^33^ First, tumor-associated neutrophils (TANs), N2-type, can promote malignancy by releasing growth-stimulating signals, matrix-degrading proteases, as well as mediators of angiogenesis. In this context, infiltration of TANs is associated with more aggressive tumors, such as the case of pancreatic adenocarcinoma.^34^ Second, high levels of N1- TANs are also associated with favorable prognosis in patients with gastric cancer.^35^ The anti-tumor activity of neutrophils has been associated with the cytotoxic capacity of neutrophils, which can create an immunological anti-metastatic barrier that prevents the establishment and growth of metastatic cells.^36^ Therefore, future studies will focus on determining the mechanism of the anti-cancer activities of donor neutrophils loaded with MYXV.

In summary, our in vitro studies illuminate a new mechanism by which both neutrophils and T cells can synergize with an oncolytic virus to target and kill residual myeloma cells. However, future in vivo studies will be needed in order to recapitulate the roles of these two leukocyte classes identified by our in vitro results. Taken together, the data presented here demonstrate that even highly “virus-resistant” residual cancer can be eliminated effectively in a novel, transplant leukocyte-dependent carrier cell model. We propose this provides potentially new therapeutic avenues to treat low levels of residual cancers in allotransplant patients following myeloablative therapy.

## Materials and Methods

### Cells and Viruses

vMyx-M135KO-GFP and vMyx-M093/Venus were described previously.^26^, ^27^ vMyx-GFP-TdTomato was described previously.^37^ Stocks of MYXV were grown on RK13 or BSC40 cells and purified by centrifugation on a sucrose cushion as previously described.^38^

MOPC315.BM cells were obtained from the laboratory of one of the authors (B.B.). Pan02 and RK13 cells were obtained from ATCC. RL5 cells were obtained from National Institutes of Health AIDS Research and Reference Reagent Program. Cultures of MOPC315.BM-DsRed- or -luciferase-expressing cells were performed as described previously^22^ with modifications as follows. In brief, cells were cultivated in RPMI 1640 GlutaMAX (GIBCO, Thermo Fisher Scientific) media supplemented with 20% heat-inactivated FBS (Atlanta Biologicals), 1X non-essential amino acids, 0.8 mg/ml geneticin (GIBCO, Thermo Fisher Scientific), and 0.005% α-thioglycerol (Sigma-Aldrich). RK13 and Pan02 cells were cultured in DMEM (Hyclone, GE Healthcare Life Sciences) supplemented with 10% heat-inactivated FBS, 292 μg/mL glutamine, 100 U/mL streptomycin, and 100 μg/mL penicillin (GIBCO, Thermo Fisher Scientific). RL5 cells were cultured in RPMI 1640 (GIBCO, Thermo Fisher Scientific) supplemented with 10% heat-inactivated FBS (Atlanta Biologicals), 100 U/mL streptomycin, and 100 μg/mL penicillin (GIBCO, Thermo Fisher Scientific).

Viral infection was performed at multiplicity of infection (MOI) of 10 ffu/cell, except where indicated otherwise. For cells grown in monolayers, growth medium was removed and infection inoculum was added in minimal volume of medium so as to cover the cells. For cells grown in suspension, cells were counted and pelleted via centrifugation. Virus was diluted in sufficient media to result in 10% of desired final volume, and cells were resuspended in virus inoculum. In all cases, adsorption of virus was performed at 37°C for 1 hr; then fresh media were added. Visualization of infection was performed by fluorescence microscopy for GFP.

### Animal Studies

All animal studies were approved by the University of Florida Institutional Animal Care and Use Committee (protocol 201405013) in strict accordance with the *Guide for the Care and Use of Laboratory Animals* of the NIH.

To establish low levels of residual murine MM, we sublethally irradiated 6- to 8-week-old BALB/c mice (Charles River Laboratories) with 175 cGy total body radiation from a Cs137 source; then mice were given 1 × 10^5^ MOPC315.BM DsRed cells suspended in PBS via tail-vein injection 24 hr later. BM transplants, derived from either age-matched C57BL/6 mice or BALB/c mice were performed 1 week later. Animals either received no transplant, 2 × 10^6^ cells of whole BM, 2 × 10^7^ ffu of vMyx-M135KO-GFP alone, or 2 × 10^6^ cells of whole BM, which had been treated ex vivo with vMyx-M135KO-GFP for 1 hr at 37°C at MOI of 10. All material was suspended in PBS and delivered by tail-vein injection. Prophylactic antibiotics were included in the water for the animals over the duration of the study to prevent secondary infection. Conditions for inclusion in longitudinal analysis required that the animal subjects survive at least to day 14 post-tumor injection, otherwise they were excluded from analysis. Animals were sacrificed at either endpoint criteria (body score of 2)^39^ or after 6 weeks. At the time of euthanasia, spleens and BM were harvested for myeloma burden analyses.

### Assessment of Myeloma Burden

Spleens were disrupted into single-cell suspensions by flushing with Hank’s balanced salt solution (HBSS) supplemented with 10% FBS followed by grinding on a 40 μM nylon cell (Thermo Fisher Scientific) strainer with a syringe plunger, and BM was disrupted by vigorous pipetting with a serological pipette. Myeloma burden was assessed by cell surface staining using anti-CD138-allophycocyanin (APC) antibody (Miltenyi Biotec) followed by flow cytometric analysis to assess percentage of total cell population that is CD138^+^, DsRed^+^. FACS data for myeloma burden were collected on a FACSCalibur flow cytometer (BD Biosciences).

### Co-cultures of Mouse C57BL/6 BM or C57BL/6 BM-Derived Cell Subpopulations with Mouse MOPC315.BM Cell Line

BM was harvested from C57BL/6 mice and subjected to red blood cells lysis using Pharm Lyse (BD Biosciences) according to the manufacturer’s instructions and counted via Cellometer Auto 2000 (Nexelcom Bioscience). Cells were either mock treated (e.g., without adding the virus) or infected with vMyx-M135KO-GFP or vMyx-GFP-TdTomato as described above. Infection was allowed to progress for 12 hr, followed by analysis using flow cytometry. BM-derived T cells were isolated via EasySep Mouse T Cell Enrichment kit (STEMCELL Technologies). BM-derived neutrophils were isolated using EasySep Mouse Neutrophil Enrichment kit (STEMCELL Technologies). T cell-depleted BM was performed using an anti-CD3ε MicroBead Kit (Miltenyi Biotec). Neutrophil-depleted BM was performed using an anti-Ly6G MicroBead Kit (Miltenyi Biotec). BM and BM-derived effector cells were infected with vMyx-M135KO-GFP as described above and mixed with target MOPC315.BM DsRed cells after virus adsorption at an effector-to-target cell ratio of 10:1. Cells were activated at the time of mixing via treatment with 70 ng/mL PMA and 2 μM ionomycin (Sigma-Aldrich) or anti-CD3/CD28 Dynabeads (Thermo Fisher Scientific). The admixtures were incubated for 72 hr followed by analysis using flow cytometry.

### Flow Cytometry to Access the Expression of Surface Proteins and to Quantify Levels of Apoptosis

FcR blocking was performed with FcR blocking reagent, mouse (Miltenyi Biotec). Antibodies used were anti-Ly-6B.2-Alexa Fluor 700 (Bio-Rad), anti-CD11b-APC, anti-Ly-6G Pacific Blue, anti-CD3-BV605, or anti-CD138-PerCP/Cy5.5 (BioLegend). Levels of apoptosis were assessed by TUNEL staining using the in situ bromodeoxyuridine (BrdU) DNA fragmentation kit (Abcam) followed by staining with anti-BrdU-APC (eBioscience) and flow cytometric analysis. Isotype controls were used accordingly: Alexa Fluor 700 Rat IgG2a, κ (clone RTK2758), APC Rat IgG2a, κ (clone RTK2758), APC Rat IgG2b, κ (clone RTK4530), BV605 Rat IgG2b, κ (clone 17A2), APC/Cy7 Rat IgG2c, κ (clone RTK4174), Pacific Blue Rat IgG2a, κ (clone RTK2758), PerCP/Cy5.5 Rat IgG2b, κ (clone RTK4530) (BioLegend). AbCTM anti-Rat/Hamster Bead kit (Molecular Probes, Thermo Fisher Scientific) was used for single-color compensation. Flow cytometry data were collected on an LSRII flow cytometer with BD FACSDiva software (BD Biosciences).

### Statistical Analyses

Log rank test or the Student’s t test was used to determine differences between different experimental groups. The reported values correspond to the mean ± SEM. A p value <0.05 was considered statistically significant.

## Author Contributions

G.M., C.C., C.L.L., and N.Y.V. proposed the scientific idea and postulated the hypotheses. C.L.L. designed and conducted the in vivo experiments. N.Y.V. and A.L.d.M. designed and conducted the in vitro experiments. G.M., C.L.L., N.Y.V, A.L.d.M., and M.M.R. interpreted the data. C.L.L., N.Y.V., and G.M. wrote the paper. H.M.A., J.-K.S.D., and T.H. provided technical support. W.C. provided the vMyx-M093/Venus construct for binding assays. B.B. provided the MOPC315.BM DsRed MM cell line for the in vitro and in vivo experiments.

## Conflicts of Interest

C.L.L. and N.Y.V. were supported by a sponsored research agreement with DNATrix. W.C. joined DNATrix during the period of this research. G.M. is a consultant for DNATrix. DNATrix only contributed to this research financially.

## Figures and Tables

**Table 1:** Fraction and Percentages of Animals in the Allograft Experiments per Treatment Cohort Scored as Disease-free, as Designated by Myeloma Burden in Spleen and BM <0.1% and No Metastases upon Necropsy

Treatment Cohort	Disease-Free/Total (n)	Percentage (%)
No transplant	5/20	25
C57BL/6 BM	3/20	15
C57BL/6 BM+MYXV	13/19	68.4

## Supplemental Information

Document S1. Supplemental Materials and Methods and Figures S1 and S2

Document S2. Article plus Supplemental Information
