# Supplementary material for: Ex Vivo Oncolytic Virotherapy with Myxoma Virus Arms Multiple Allogeneic Bone Marrow Transplant Leukocytes to Enhance Graft versus Tumor
Source: Mol Ther Oncolytics. 2016 Dec 14;4:31–40. doi: 10.1016/j.omto.2016.12.002 (PMC5363758; doi:10.1016/j.omto.2016.12.002)
Supplement: Document S1. Supplemental Materials and Methods and Figures S1 and S2 [file mmc1.pdf]

OMTO, Volume *4*

Supplemental Information

Ex Vivo Oncolytic Virotherapy with Myxoma Virus Arms Multiple Allogeneic Bone Marrow Transplant Leukocytes to Enhance Graft versus Tumor

Cameron L. Lilly, Nancy Y. Villa, Ana Lemos de Matos, Haider M. Ali, Jess-Karan S. Dhillon, Tom Hofland, Masmudur M. Rahman, Winnie Chan, Bjarne Bogen, Christopher Cogle, and Grant McFadden

SUPPLEMENTARY MATERIAL

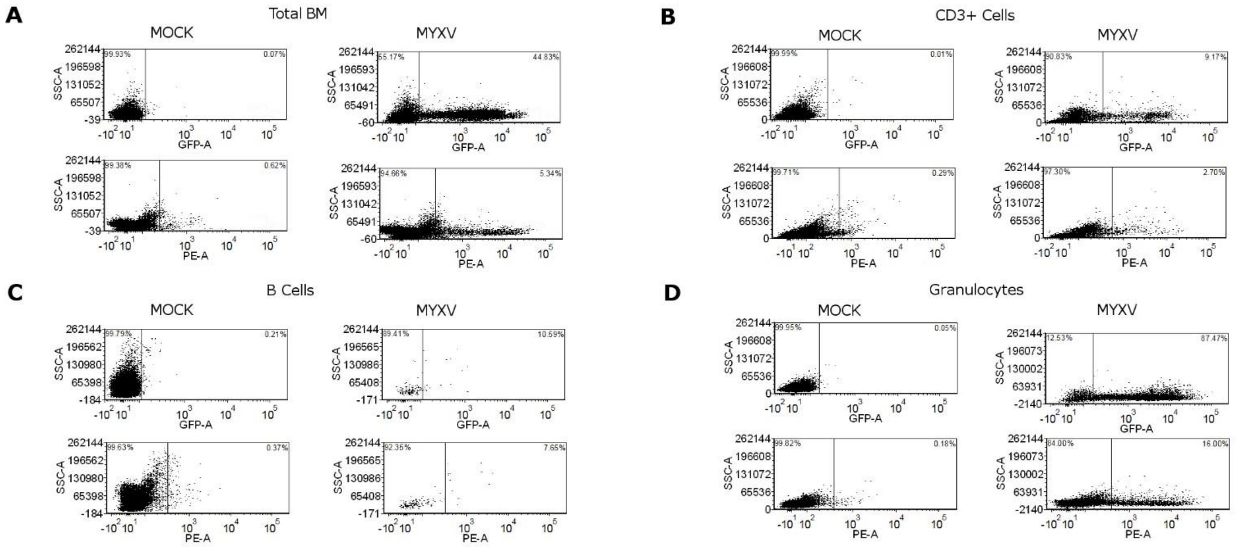

Figure S1. Expression of late viral genes by different murine bone marrow cell types

To determine the proportion of infected GFP^+ cells that become TdTomato positive (indicating the progression to late viral gene expression), murine bone marrow was infected with vMyxGFP/TdTomato at MOI of 10. Panel (**a**) represents whole BM. BM-derived T cells (**b**) were analyzed by CD3 staining. Within the infected BM, B cells were identified by B220/CD19 staining (**c**), and granulocytes were identified as being $CD11b^+$/Ly-$6B.2^+$/Ly-$6G^{hi}$ (**d**).

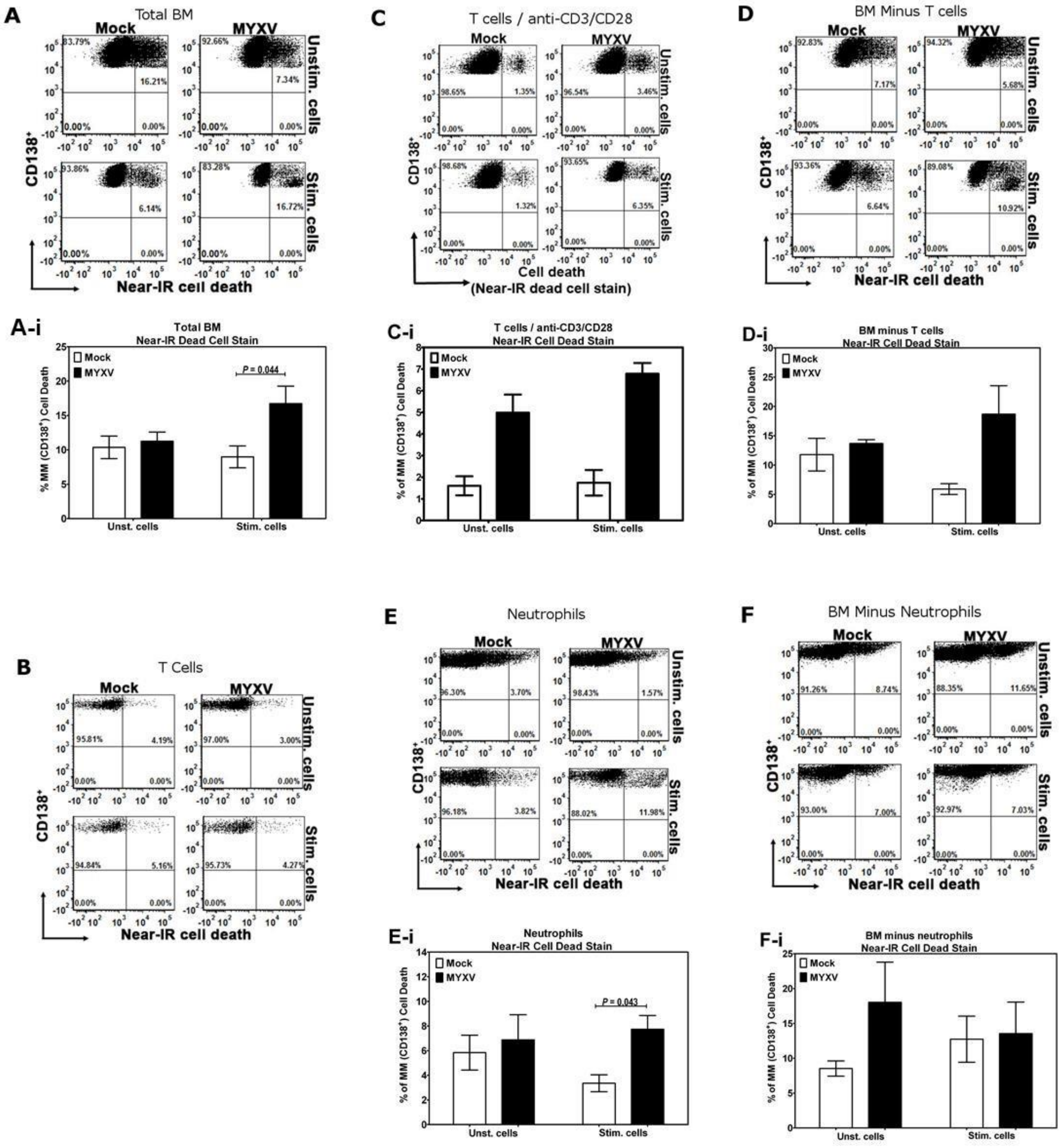

Figure S2. MOPC315.BM cell death induced by effector cells armed with myxoma virus

NearIR Live/Dead® staining and flow cytometric analysis were performed on MLRs combining mock- or MYXV-treated, unstimulated or stimulated C57BL/6 BM-derived cells (effector) with MOPC315.BM DsRed (target) at a ratio of 10:1. MLRs were performed with whole BM (**a and a-i**), negatively selected T cells (**b**, **c and c-i**), or T cell depleted cell fraction (**d and d-i)**,

negatively selected neutrophils (**e and e-i**), and neutrophil depleted (**f and f-i**). The mean of at least three independent experiments is shown as the height of the bar and error bars represent the Standard Error (SE). *P* values were determined using the Student's t-test. A *P* value of less than 0.05 was considered statistically significant.

SUPPLEMENTAL METHODS

Progression of virus infection of different murine bone marrow cell types

Levels of expression of late viral genes was performed using vMyx-GFP/TdTomato recombinant virus. In brief BM from C57BL/6 mice was infected with vMyx-GFP (Early/Late promoter)TdTomato (Late promoter) at an MOI of 10 for 12 hr. After blocking with mouse-specific FcR reagent, (Miltenyi Biotec Inc., San Diego, CA), cells were stained with antibodies against anti-Ly-6B.2-Alexa Fluor® 700 (Bio-Rad, Hercules, CA), anti-CD11b-APC, anti-Ly-6G Pacific Blue, anti-B220-APC, anti-CD19-Pacific Blue, anti-CD3-APC/Cy7 and analyzed using flow cytometry for GFP^+ or PE^+ cells. Isotype controls were used accordingly: Alexa Fluor® 700 Rat IgG2a, κ (clone RTK2758), APC Rat IgG2a, κ (clone RTK2758), APC Rat IgG2b, κ (clone RTK4530), APC/Cy7 Rat IgG2b, κ (clone RTK4530), APC/Cy7 Rat IgG2c, κ (clone RTK4174), Pacific Blue Rat IgG2a, κ (clone RTK2758). AbCTM anti-Rat/Hamster Bead kit (Molecular Probes™, Thermo Fisher Scientific, Waltham, MA) was used for single color compensation.

Assessment of MOPC315.BM cell death induced by effector cells armed with myxoma virus

Sample viability was assessed using the Live/Dead® Fixable Blue Dead Cell Stain kit, for UV excitation or Live/Dead® Fixable Near-IR Dead Cell Stain Kit, for excitation with 633 or

635 nm (Molecular Probes™, Thermo Fisher Scientific, Waltham, MA). FcR blocking was performed with mouse specific FcR reagent (Miltenyi Biotec Inc., San Diego, CA). Cells were then stained with anti-CD138-PerCP/Cy5.5 antibody (BioLegend, San Diego, CA) or with the isotype control PerCP/Cy5.5 Rat IgG2b, κ (clone RTK4530) (BioLegend, San Diego, CA). AbCTM anti-Rat/Hamster Bead kit (Molecular Probes™, Thermo Fisher Scientific, Waltham, MA) was used for single color compensation. Levels of CD138 cell death were quantified using flow cytometry.
